# HOXB13 is downregulated in colorectal cancer to confer TCF4-mediated transactivation

**DOI:** 10.1038/sj.bjc.6602631

**Published:** 2005-05-31

**Authors:** C Jung, R-S Kim, H Zhang, S-J Lee, H Sheng, P J Loehrer, T A Gardner, M-H Jeng, C Kao

**Affiliations:** 1 Department of Urology, Indiana University, Indianapolis, IN 46202, USA; 2 Department of Surgery, Indiana University, Indianapolis, IN 46202, USA; 3 Department of Medicine, Indiana University, Indianapolis, IN 46202, USA; 4 Department of Microbiology and Immunology, Indiana University, Indianapolis, IN 46202, USA

**Keywords:** HOXB13, homeobox gene, TCF4, colorectal cancer

## Abstract

Mutations in the Wnt signalling cascade are believed to cause aberrant proliferation of colorectal cells through T-cell factor-4 (TCF4) and its downstream growth-modulating factors. HOXB13 is exclusively expressed in prostate and colorectum. In prostate cancers, HOXB13 negatively regulates *β*-catenin/TCF4-mediated transactivation and subsequently inhibits cell growth. To study the role of HOXB13 in colorectal tumorigenesis, we evaluated the expression of HOXB13 in 53 colorectal tumours originated from the distal left colon to rectum with their matching normal tissues using quantitative RT–PCR analysis. Expression of HOXB13 is either lost or diminished in 26 out of 42 valid tumours (62%), while expression of TCF4 RNA is not correlated with HOXB13 expression. TCF4 promoter analysis showed that HOXB13 does not regulate TCF4 at the transcriptional level. However, HOXB13 downregulated the expression of TCF4 and its target gene, c-myc, at the protein level and consequently inhibited *β*-catenin/TCF-mediated signalling. Functionally, forced expression of HOXB13 drove colorectal cancer (CRC) cells into growth suppression. This is the first description of the downregulation of HOXB13 in CRC and its mechanism of action is mediated through the regulation of TCF4 protein stability. Our results suggest that loss of HOXB13 may be an important event for colorectal cell transformation, considering that over 90% of colorectal tumours retain mutations in the APC/*β*-catenin pathway.

Colorectal cancer (CRC) is the third most common cancer in the United States, with an estimated 146 940 new cases in 2004, including 106 370 patients with colon cancer and 40 570 with rectal cancer (Jemal *et al*, 2004). Cancers of the proximal colon account for 41.2% of these cases, the descending colon 4.7%, and the distal colon and rectum 49.3% (Cheng *et al*, 2001). Metastatic CRC is the second leading cause of death from cancer in North America, largely due to the poor clinical response of tumours to conventional chemotherapeutics.

Mutations in the Wnt signalling pathway are frequently connected to aberrant proliferation of colon cells. Over 80% of CRC (Bienz and Clevers, 2000; Polakis, 2000) have mutations of adenomatous polyposis coli (*APC*) gene. APC complexes with Axin and GSK3*β* to phosphorylate *β*-catenin, which will be degraded. Mutated APC leads *β*-catenin to accumulate and consequently translocate into the nucleus. In a significant fraction of sporadic CRC lacking APC mutations, constitutively active mutations were found in *β*-catenin gene and removes target residues of this kinase. Rare mutations in Axin have also been found in colon tumours. Taken together, all these mutations lead to the activation of members of the T-cell factor (TCF) family transcription factor, resulting in target gene transcription.

*HOX* homeobox genes regulate axial regional specification during embryonic development and are often expressed in a tissue-specific and stage-related manner. *Hoxb13* is limitedly expressed in the caudal extent of the spinal cord, tail bud and urogenital sinus (Zeltser *et al*, 1996; Sreenath *et al*, 1999). In the adult mouse, *Hoxb13* is confined to the epithelial cells of the prostate and distal colon and rectum (Zeltser *et al*, 1996; Sreenath *et al*, 1999). Tissue-specific expression of HOXB13 is also well reported in human (Aarnisalo *et al*, 1998; Edwards *et al*, 2005; Hood *et al*, 2004; Jung *et al*, 2004b; Takahashi *et al*, 2004). The loss-of-function Hoxb13 mouse manifests inhibition of proliferation and activation of programmed cell death in the secondary neural tube and caudal somites (Economides *et al*, 2003). Recently, we demonstrated that the forced expression of HOXB13 suppressed the growth of metastatic prostate cancer cells by disrupting *β*-catenin/TCF signals (Jung *et al*, 2004a). In particular, HOXB13 inhibited the expression of TCF4 and subsequently suppressed the expression of TCF-responsive genes, including c-myc and cyclin D1. However, expression of HOXB13 was not altered during prostate tumorigenesis. In this study, we investigated the expression of *HOXB13* in colorectal tumours and the involvement of HOXB13 in the *β*-catenin/TCF signalling pathway.

## MATERIALS AND METHODS

### Reagents

pFLAG-HOXB13, pIRES-HOXB13, and Ad-GFP-HOXB13 were described previously (Jung *et al*, 2004a). pIRES-TCF4RE-luc contains four copies of the TCF4 response elements derived from cyclin D1 promoter as also described. pBV-c-myc-luc was generously provided by Dr Bert Vogelstein. pCMV-*β*-gal was from Promega (Madison, WI, USA). To construct pTCF4-luc, a potential promoter region encompassing ∼1.9 kb upstream of the transcription start site was predicted, acquired from human BAC clone (clone number RP11-57H14 from the library RPCI-11.1; BACPAC Resources, Oakland, CA, USA), and PCR-cloned into pGL-luciferase system. Anti-FLAG M5 and anti-c-myc antibodies came from Sigma (St Louis, MO, USA) and BD Biosciences (Palo Alto, CA, USA), respectively. Monoclonal antibodies to TCF4 and transcription factor IID (TFIID) came from Upstate Biotechnology (Charlottesville, VA, USA). Anti-*β*-catenin was from Santa Cruz Biotechnology (Santa Cruz, CA, USA).

### Cell cultures

Selected human CRC cells were purchased from ATCC and propagated according to the company's instructions. These include HCT116, HT-29, SW480, LoVo, Caco-2, SW837, SW1463, and NCI-H630. LNCaP human prostate cells were also cultured in RPMI media (Invitrogen, Carlsbad, CA, USA) supplemented with 5% FBS at 37°C in an atmosphere containing 5% CO_2_ as described previously (Wu *et al*, 1998). All cultures were fed with fresh medium every 3–4 days.

### RNA sample preparation

Cells were grown up to 80% confluency before RNA extraction. RNA from normal rectum was purchased from two other sources (BD Biosciences, Palo Alto, CA, USA and Stratagene, La Jolla, CA, USA) to reduce sampling errors. RNA from LNCaP prostate cancer cells was used for standardising the expression levels of HOXB13 and *β*-actin, an internal control. Surgical frozen specimens of human colon cancer were acquired from the Indiana University Tissue Bank. Collected tumour samples originated from rectum or sigmoid colon included their adjacent normal colon tissues. Samples were homogenated in PBS using Tissue Homogenizer (Kinematica, Switzerland). Total RNAs were purified from both cultured cells and surgical specimen using Ultraspec RNA isolation system (Biotecx Laboratories Inc., Houston, TX, USA) as described previously (Jung *et al*, 2001). Each RNA (1 *μ*g) was reverse-transcribed using dT(15) primers and Superscript II (Invitrogen, Carlsbad, CA, USA). No reverse transcriptase controls were run together to identify genomic DNA contamination. RNAs were prepared again once the contamination was found.

### Quantitative RT–PCR (QRT–PCR) analysis

QRT–PCR analysis was performed using the iCycler system (BioRad, Hercules, CA, USA) with the *Quantitect* SYBR Green PCR kit (Qiagen, Valencia, CA, USA) according to the manufacturer's instructions. *HOXB13* primers (5′-ccccactgagtttgccttctatc-3′, 5′-gcctcttgtccttggtgatgaac-3′) are designed to amplify specifically a coding region of 369–739. TCF4 primers were 5′-tcccaccacatcatacgctacac-3′ and 5′-tcgcttgctcttctctggacag-3′. Primers for *β*-actin were used to show the equal loading (5′-gcaccacaccttctacaatgagc-3′, 5′-tagcacagcctggatagcaacg-3′). cDNA (50 ng) were used in a final reaction volume of 25 *μ*l. Standard curves were prepared from serial dilutions of LNCaP cDNA. A water blank was also incorporated in each run on 96-well optical reaction plates. PCR cycling conditions were performed for all of the samples as follows: 10 min at 95°C for enzyme activation; 40 cycles for the melting (95°C, 15 s): and annealing/extension (30 s) steps. To assess the specificity of each primer set, amplicons generated from the PCR reaction were analysed by their melting point curves and additionally run on 1% agarose gels to confirm the correct sizes of the PCR products. Using the standard curve method generated by amplification of 0.08–50 ng of LNCaP cDNA, the resulting *C*_t_ values were converted to picogram quantities. Then, the *HOXB13* quantity was normalised by *β*-actin and subtracted from no reverse transcriptase controls. This value was then averaged for each duplicate. Each sample was prepared in duplicate and the experiment was repeated once to increase the power analysis.

Statistical analysis was performed using GraphPad Prism software (San Diego, CA, USA). First, the mean expression level of HOXB13 was acquired from 38 valid normal tissues out of 42 samples analysed. Four normal samples did not show RNA integrity as seen by *β*-actin amplification. Then, a 95% confidence interval was obtained. Differences in the measured expression of HOXB13 mRNA between normal and tumour groups were analysed by Student's *t*-test. A *P*-value of less than 0.05 was considered statistically significant.

### Transient transfections

Approximately 1 × 10^5^ cells were plated in a 24-well plate 16 h before transfection. Transfections were carried out using Lipofectamine 2000 (Invitrogen) with 0.1 *μ*g of reporter, 0.1 *μ*g of test plasmid, and 20 ng of pCMV-*β*-gal as described by the manufacturer's protocol. At 6 h after transfection, the cells were washed and fed with medium containing 5% FBS. After 18 h, cells were washed with PBS, lysed with 100 *μ*l of passive lysis buffer, and assayed for luciferase activity as relative light units using Luciferase assay system (Promega). *β*-Galactosidase activity was assessed for the normalisation and transfection efficiency. Lysates (15 *μ*l) were incubated in 150 *μ*l of ONPG solution (1 mM MgCl_2_, 10.8 mg ml^−1^ *o*-nitrophenyl-*β*-D-galactopyranoside, 0.1 M sodium phosphate, 45 *μ*M *β*-mercaptoethanol) at 37°C for 30 min. Reactions were stopped by the addition of 200 *μ*l of 1 M Na_2_CO_3_, and activity was measured as absorbance at 420 nm. Transfection experiments were carried out in triplicate and results were reported as mean±s.d.

### Western blotting assay

Cells were grown up to 80% confluence in corresponding media containing 5% FBS. Cells were harvested, lysed with lysis buffer (10 mM HEPES (pH 7.9), 1.5 mM MgCl_2_, 10 mM KCl, 0.5 mM DTT, 0.5 mM PMSF), and incubated on ice for 10 min. Nuclei from the cell lysate were pelleted by microcentrifugation and resuspended in nuclear extraction buffer (20 mM HEPES (pH 7.9), 420 mM NaCl, 1.5 mM MgCl_2_, 0.5 mM EDTA, 10 mM KCl, 0.5 mM DTT, 0.5 mM PMSF, 25% glycerol). Cell debris was removed by centrifugation at 4°C for 5 min. In all, 20 *μ*g of nuclear extracts were loaded onto 10% Bis–Tris gel (Invitrogen) and separated using the Novex electroporation system (Invitrogen). After proteins were transferred to PVDF membrane, primary antibodies were applied, followed by incubation with horse peroxidase-conjugated secondary antibodies. Blots were developed by the ECL detection system (Pierce, Rockford, IL, USA).

### Growth assays

Selected CRC cells, including HCT116, Caco-2, LoVo, and SW480, were monitored under the fluorescence microscope after virus infection to see the functional effect of HOXB13. Briefly, cells were infected with either Ad-GFP or Ad-GFP-HOXB13 at a multiplicity of infection (MOI) of 2. Every 3–4 days, fresh media were added. An MTT *in vitro* proliferation assay was also performed as described previously (Jung *et al*, 2004a). Briefly, cells were plated onto a 96-well plate and infected with either Ad-GFP-HOXB13 or Ad-GFP (1 MOI). At days 1–7 after virus infection, cells were stained with 25 *μ*l of 5 mg ml^−1^ MTT solution and incubated for 2 h at 37°C. The reaction was stopped by adding 100 *μ*l of extraction buffer (50% formamide and 10% SDS, pH 4.7). After overnight incubation at 37°C, the absorbance at 570 nm was measured using a Spectra Microplate Reader (Molecular Devices, Sunnyvale, CA, USA). Densitometric values were analysed with Student's *t*-test.

## RESULTS

### Expression of *HOXB13* in colorectal tumours

**Figure 1:**
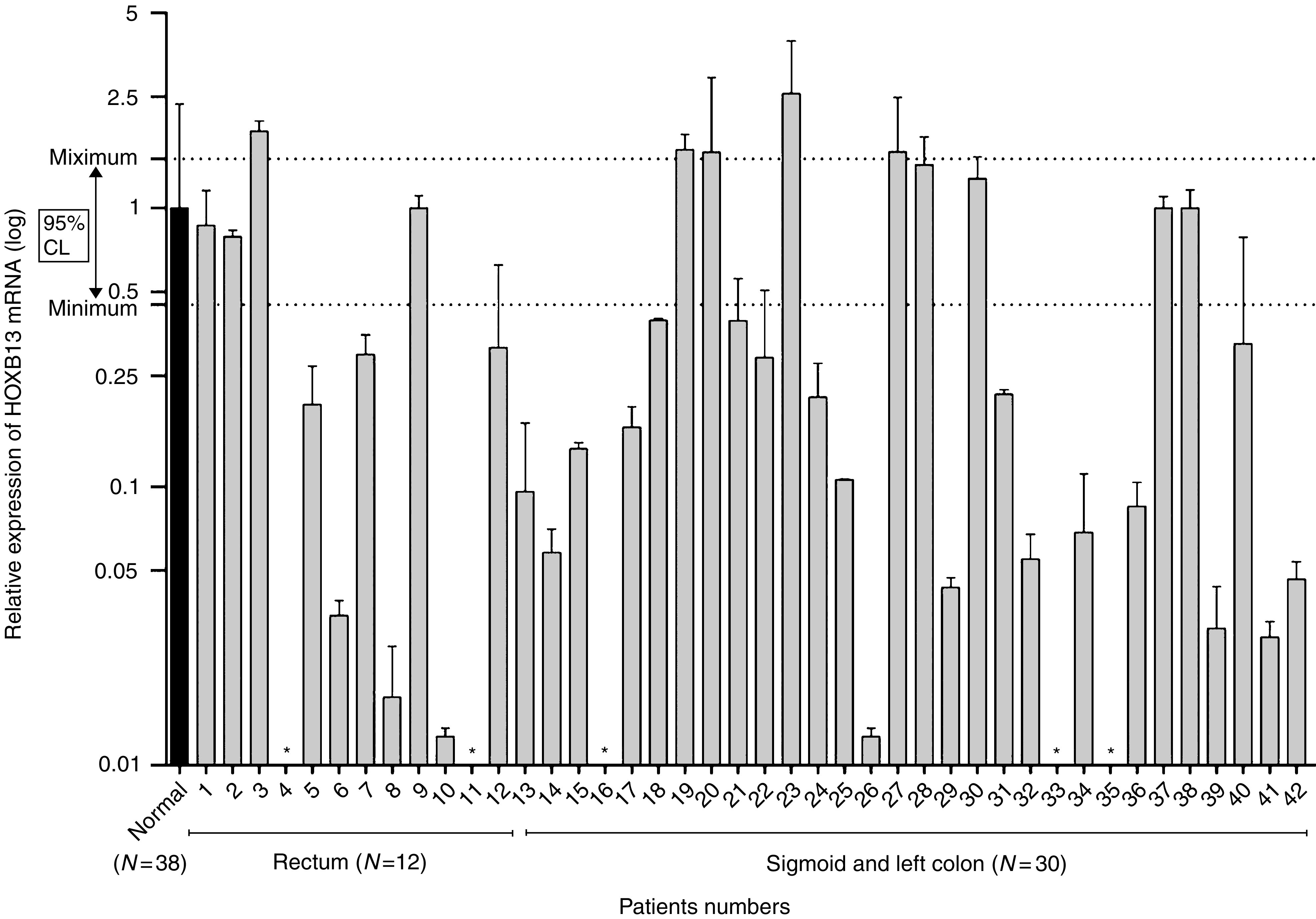
Expression pattern of *HOXB13* mRNA in colorectal tumours. Real-time RT–PCR was performed with RNA extracted from colorectal tumours and matching normal tissues. Levels of *HOXB13* in both normal and tumour tissues were normalised by *β*-actin. Relative expression of *HOXB13* in normal tissues were averaged (black bar), which was used for comparative HOXB13 expression in each tumours. Dotted line represents 95% confidence interval for the range of *HOXB13* expression in normal colorectum. ^*^HOXB13 not detectable in tumours. Note X-axis is a log scale.

To correlate the expression of *HOXB13* and colorectal tumorigenesis, QRT–PCR was performed in 53 colorectal tumours. In mice, expression of *Hoxb13* is restricted to rectum and distal region of colon (Sreenath *et al*, 1999). Samples were tumours anatomically originating from descending colon to rectum, consisting of nine distal left colons, 31 sigmoid colons, and 13 rectums. Each tumour sample was accompanied by adjacent matching normal tissues. Total RNA was extracted from the samples followed by real-time RT–PCR analysis using SYBR Green. *β*-Actin was used to normalise samples and to quantitate RNA from epithelial cells. We then calculated relative amounts of mRNAs by the standard curve method in relation to *β*-catenin levels. Out of 53 samples, 11 samples were eliminated because of (1) the lack of RNA preservation in repeated experiments evidenced by *β*-actin expression in tumour tissues, (2) no *HOXB13* expression in normal matching tissues, leading us to suspect that origin of tumours are anatomically HOXB13-free region, especially in distal colon. Melt-curve analysis was also performed to eliminate nonspecific amplification as well as the contribution from primer dimer formation. There was no substantial difference in expression level of *HOXB13* between each anatomic region in the normal tissues. The expression level of *HOXB13* from all normal tissues was averaged to acquire a relative expression level of *HOXB13* for each tumour. As shown in Figure 1, expression of *HOXB13* mRNA is diminished in 26 out of 42 CRC (62%). Five showed complete loss of *HOXB13* mRNA. There was no correlation between the loss of *HOXB13* and the origin of the tissues. Previously, we have observed that HOXB13 negatively regulates the expression of TCF4 in prostate cancer cells. Therefore, we performed QRT–PCR using TCF4-specific primers to see if *HOXB13* inversely correlates to TCF4. However, there was no inverse correlation between HOXB13 and TCF4 at the RNA level (data not shown).

### HOXB13 inhibits *β*-catenin/TCF-mediated transactivation

**Figure 2:**
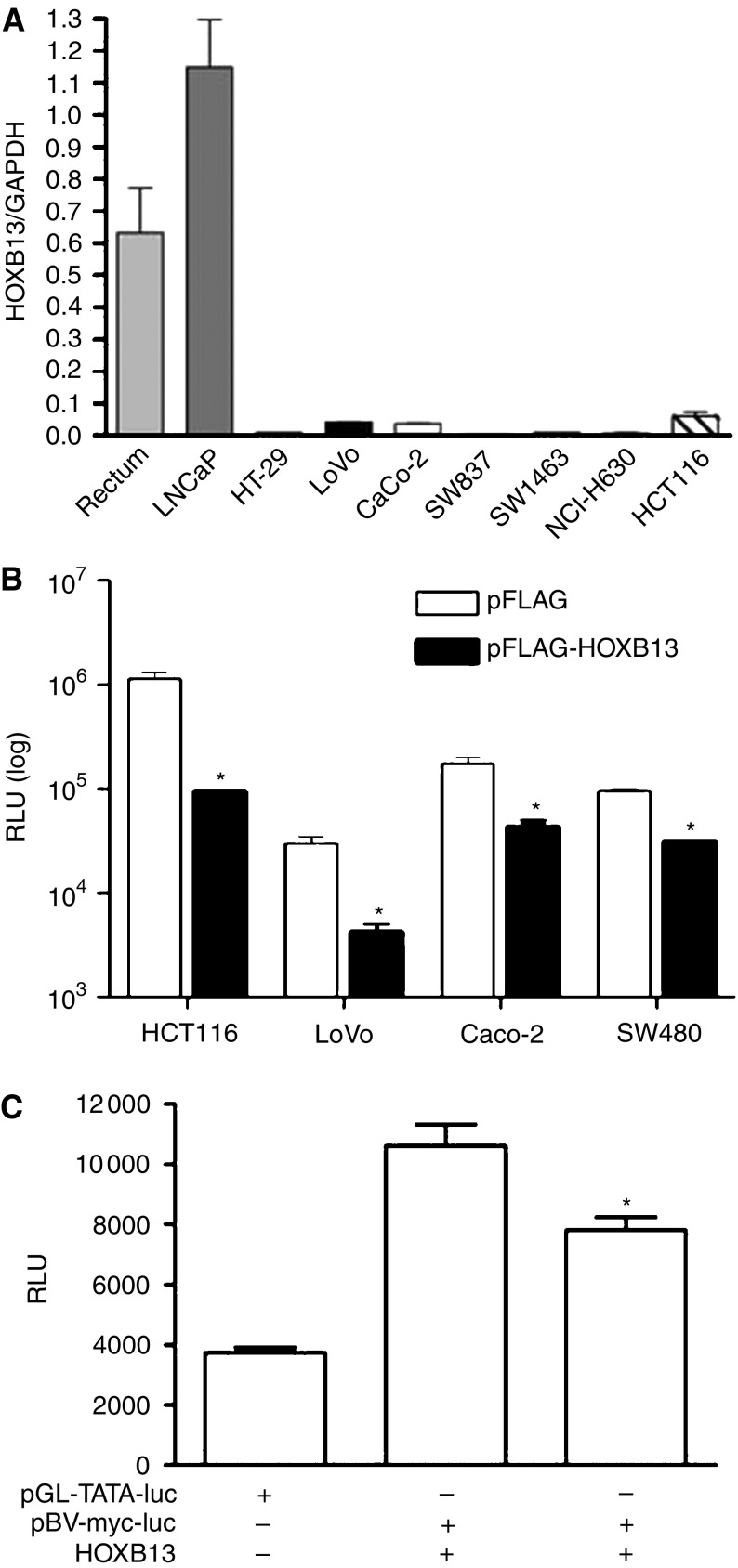
*HOXB13* disrupts *β*-catenin/TCF-mediated signals. (**A**) Real-time RT–PCR was performed to show baseline levels of *HOXB13* in established colorectal tumour cell lines. Normal rectum and LNCaP prostate cancer cells were used as references. (**B**) Cells were transiently transfected with 100 ng of pIRES-TCF4RE-Luc, 20 ng of pCMV-*β*-gal, and 100 ng of pFLAG-HOXB13 with or without 10 nM R1881. pFLAG-CMV was used as a counterpart of pFLAG-HOXB13. ^*^P<0.05. (**C**) CV-1 cells were transfected with either pGL-TATA-luc or pBV-myc-luc and pIRES-HOXB13. ^*^P=0.02.

**Figure 3:**
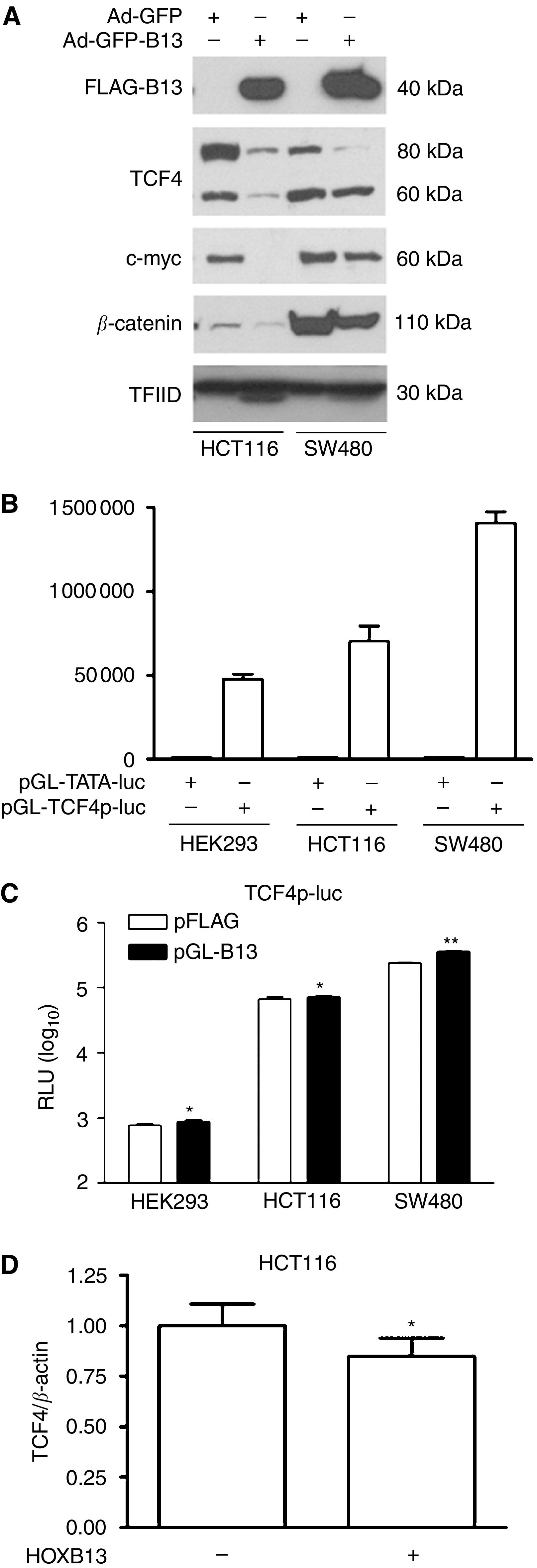
*HOXB13* suppresses expression of TCF4 at the post-transcriptional level. (**A**) Cells were infected with either Ad-GFP or Ad-GFP-HOXB13 virus (100 MOI) for 48 h. Nuclear proteins were separated by gel electrophoresis followed by Western blot analysis. (**B**) Potential promoter region of TCF4 was cloned into pGL-luc system (pGL-TCFp-luc) followed by transient transfection assay. (**C**) Cells were transfected with pGL-TCF4p-luc and either pFLAG or pFLAG-HOXB13. ^*^P>0.2; ^**^P<0.05. (**D**) HCT116 cells were infected with Ad-GFP or Ad-GFP-HOXB13 (100 MOI) followed by extraction of total RNA. Real-time RT–PCR was performed for TCF4 expression and evaluated by standard curve methods. RNA from normal rectum was used to draw the standard curve for TCF4 and *β*-actin. ^*^P=0.32.

In order to investigate the function of HOXB13, baseline expression level of HOXB13 was first determined in selected CRC cells by QRT–PCR. As shown in Figure 2A, all CRC cells did not express significant amounts of *HOXB13* compared to normal rectum and LNCaP prostate cancer cell. Note that SW837, SW1463, and NCI-H630 are rectum-oriented cancer cells. This result suggests that loss of *HOXB13* is also seen in all CRC cells. Next, we investigated whether HOXB13 is involved in the regulation of *β*-catenin-TCF-regulated transcription (CRT), which is constitutively activated in most colorectal tumours. All the CRC cells used in this study retain either truncated APC or mutation(s) in *β*-catenin phosphorylation site, causing constitutive activation of CRT (Morin *et al*, 1997; Shih *et al*, 2000). We employed artificial promoter reporter system containing four copies of the TCF4 response elements derived from cyclin D1 promoter. Figure 2B demonstrates that the overexpression of HOXB13 greatly inhibited TCF4 response in all CRC cells tested. Most noticeably, HOXB13 decreased CRT activity up to 12-fold in HCT116 cells, in which *β*-catenin has a deletion of Ser^45^ inhibiting the downregulation of *β*-catenin through phosphorylation. To avoid the possible misinterpretation from using an artificial promoter, we studied the effect of HOXB13 in natural c-myc promoter linked to luciferase. c-Myc is also known to be a TCF4-responsive gene. For higher transfection efficiency, CV-1 monkey kidney cells were cotransfected with HOXB13 (Figure 3C). HOXB13 moderately suppressed the promoter activity of c-myc (20%, *P*=0.02).

### HOXB13 suppresses the expression of TCF4 at the translational level

HOXB13 (100 MOI) was transduced into selected CRC cells via adenoviral vector to learn how HOXB13 regulates the expression of TCF4 and its responsive gene, c-myc. Western blot analysis demonstrated that forced expression of HOXB13 dramatically decreased the expression of TCF4 protein in HCT116 and SW480 cells (Figure 3A). As observed in prostate cancer cells by another group (Chesire *et al*, 2002), two isoforms of TCF4 proteins (∼80 and 60 kDa) were detected in CRC cells. The 80 kDa TCF4 isoform is SUMO-modified and likely contribute to the majority of TCF4 activity. In our study, effect of HOXB13 was greater on the 80 kDa isoform than the 60 kDa isoform. Accordingly, expression of c-myc protein was significantly suppressed by HOXB13, especially in HCT116 cells. While minimal suppression of *β*-catenin was also observed, HOXB13 did not affect the expression of TFIID.

To see how HOXB13 is involved in the regulation of TCF4 expression, the promoter region of TCF4 was explored and tested for the effect of HOXB13. Owing to a lack of information on the TCF4 promoter region (Duval *et al*, 2000), a potential promoter region encompassing ∼1.9 kb upstream of the ATG site was acquired from human BAC clone (clone number RP11-57H14 from the library RPCI-11.1; BACPAC Resources, Oakland, CA, USA) and PCR-cloned into pGL-luciferase system. Resulting pGL-TCF4p-luc was transfected into HEK293, HCT116, and SW480 cells followed by luciferase assay. The promoter region had almost 150-fold transcriptional activity compared to pGL-TATA-luc control (Figure 3B). To see the effect of HOXB13 on this TCF4 promoter region, pFLAG-HOXB13 was cotransfected into the cells. However, expression of HOXB13 did not inhibit the TCF4 promoter activity in all cells tested (Figure 3C). Instead, HOXB13 slightly increased the promoter activity of TCF4 in SW480 cells (*P*<0.05), while HOXB13 did not affect both HEK293 and HCT116 cells (*P*>0.05). Consequently, overexpression of viral HOXB13 did not alter the expression of TCF4 mRNA in HCT116 cells, as demonstrated by QRT–PCR (Figure 3D). These results suggest that suppression of TCF4 by HOXB13 occurs at the post-transcriptional level and may explain why RNA expression of HOXB13 and TCF4 was not correlated in patient tumour samples.

### HOXB13 inhibits the growth of CRC cells

**Figure 4:**
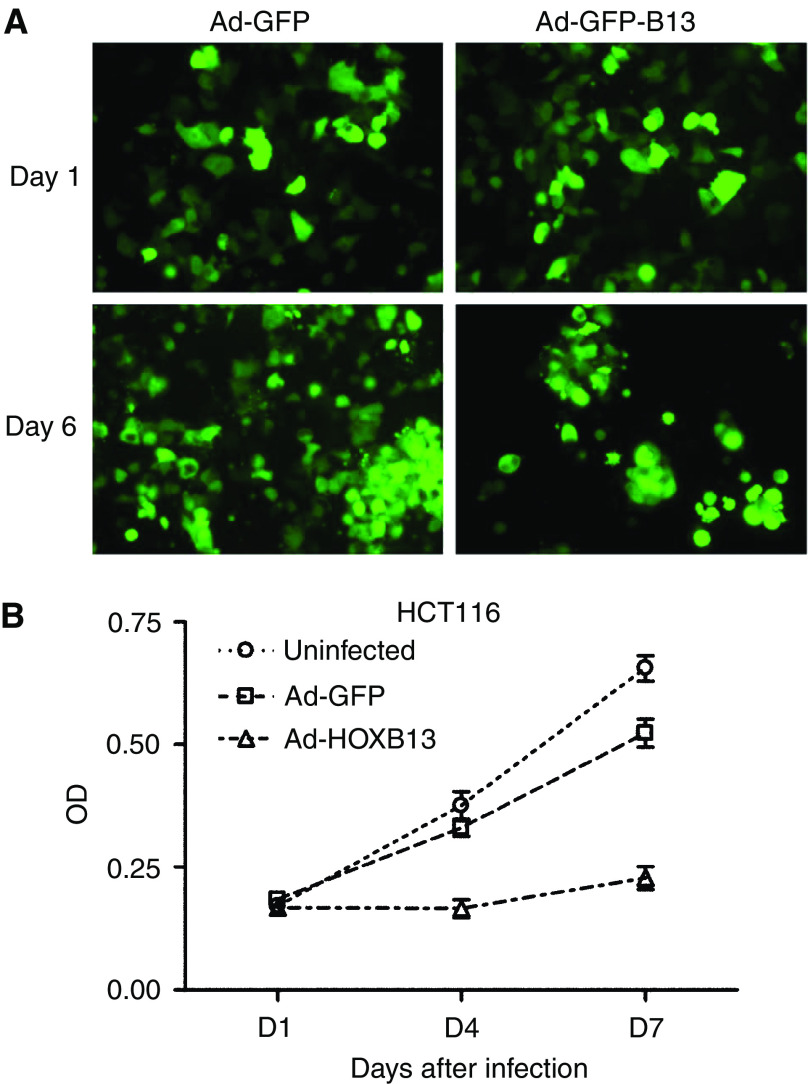
*HOXB13* inhibits the proliferation of HCT116 colon cancer cells. (**A**) Ad-HCT116 cells were infected with either Ad-GFP-HOXB13 or Ad-GFP viruses (10 MOI). GFP-expressing cells were monitored under the fluorescence microscope. (**B**) The same study was performed as in (**A**). At indicated days after virus infection (1 MOI), HCT116 cells were stained with MTT reagent and optical density at 570 nm was measured.

In order to see if HOXB13 regulates the growth of CRC cells, cells were infected with recombinant adenovirus expressing both GFP and HOXB13 (10 MOI). Ad-CMV-GFP was used as a control virus. Figure 4A demonstrated that HOXB13 suppressed the growth of HCT116 cells 6 days after infection. HOXB13 also inhibited the growth of LoVo, SW480, and Caco-2 (data not shown). The growth-suppressive function of HOXB13 was confirmed by MTT *in vitro* proliferation assay using HCT116 cells. First, cells (2 × 10^3^) were plated onto 96-well plates and viruses were administered as described above (1 MOI). At each indicated days, cells were lysed and checked for OD for live cells. Figure 4B showed that HOXB13 dramatically decreased the proliferation of HCT116 cells compared to control virus-infected cells.

These results suggest that HOXB13 functions as a growth suppressor in CRC cells and the loss of HOXB13 may be an important event for the CRC development. They also imply that HOXB13's growth suppressive function is, at least in part, accomplished through the disruption of *β*-catenin/TCF signalling, which is aberrantly activated in most of CRC.

## DISCUSSION

Although HOX proteins have long been known to be important regulators in proper embryonic development in a temporal and spatial expression pattern, little is known about their involvement in solid tumour formation. In addition, expression of some HOX proteins is maintained during the adult stage without their clear biological roles. HOXB13 is specifically, if not exclusively, expressed in prostate and colorectum. In this study, we demonstrated that downregulation of HOXB13 is a common event during the colorectal tumorigenic process. Edwards *et al* (2005) have recently shown that HOXB13 was markedly downregulated in tumour compared to normal colon in their cDNA microarray experiment despite limited number of tumour samples. We limited patient tumour samples to those from the descending colon to rectum since the expression of *HOXB13* is known to be restricted to the distal colon and rectum by both mouse and human studies (Sreenath *et al*, 1999; Jung *et al*, 2004b). HOXB13 seems to function as a growth suppressor by negatively regulating signals from the CRT pathway, which is usually aberrantly activated in colorectal tumours. Overexpression of HOXB13 inhibited the expression of TCF4 protein, not its RNA, implying that HOXB13 mediates post-transcriptional reduction of TCF4 levels by an unknown mechanism. This may explain why HOXB13 did not inversely correlate with the expression of TCF4 in patient tumour samples in our RNA analyses. Owing to the lack of antibodies against HOXB13, extensive further study is needed. Nevertheless, we propose that the loss of HOXB13 may be an additional important event for CRC cells, conferring a growth advantage to fully utilise limited TCF4 and subsequently overexpress its downstream genes.

TCF4 is the most abundantly expressed TCF in colorectal epithelium (Korinek *et al*, 1998) and directly regulates the transcription of c-myc (He *et al*, 1998), cyclin D1 (Tetsu and McCormick, 1999), and the metalloproteinase matrilysin (MMP-7) (Brabletz *et al*, 1999), whose expression is thought to be the basis for tumorigenesis. In fact, TCF4 knockout mice have complete loss of stem cells in the small intestine (Korinek *et al*, 1998). c-Myc was identified as a target of the Wnt signalling pathway, with its expression being repressed by wild-type APC and activated by *β*-catenin via inappropriate complex formation with TCF4 (He *et al*, 1998). It was shown that c-myc is overexpressed in early and late stages of CRC (Sikora *et al*, 1987; Smith *et al*, 1993). In present study, we demonstrated that HOXB13 suppressed the expression of this powerful oncogene by possibly suppressing TCF4 expression. In other words, loss of HOXB13 may stimulate the expression of c-myc to help the colorectal tumour development. Compared to HCT116 cells, SW480 cells showed much less downregulation of c-myc protein expression in response to HOXB13 overexpression. As described by others (Korinek *et al*, 1997; He *et al*, 1998), this is possibly due to low-level expression of TCF4 in SW480 cells, suggesting that other TCF/LEF isoforms are more involved in CRT pathway.

In the prostate, we have demonstrated that HOXB13 negatively regulates two important growth signals, CRT (Jung *et al*, 2004a) and hormone-activated androgen receptor (AR)-mediated transcription (Jung *et al*, 2004b). Depending on the status of AR, suppression of either pathway by HOXB13 seems to inhibit the growth of prostate cancer cells. Deregulation of CRT by HOXB13 results from the downregulation of TCF4 transcription factors, although aberrant activation of CRT pathway is seen in only small fractions of prostate tumours (Voeller *et al*, 1998; Chesire *et al*, 2000). In prostate cancer, however, TCF4 is regulated at the transcription level while at the translational suppression in CRC without known mechanism(s). It is conceivable that there may be a difference in cell-to-cell response. Otherwise, several isoforms of TCF4 were not differentiated by whole promoter analysis applied in this study. Recently, there are increased evidences on communication between AR and *β*-catenin/TCF signalling. In fact, AR physically interacts with both *β*-catenin (Song *et al*, 2003) and TCF4 (Amir *et al*, 2003), suggesting that AR may be deeply involved in the function of HOXB13 as a modulator to control these two important signalling pathways. We suppose that AR has a protective mechanism against HOXB13-induced growth suppression by relieving HOXB13 effect on CRT or simply activating AR-mediated signals. It is also conceivable that loss of HOXB13 will lead tumour development and/or tumour progression to advanced form. In fact, CRT pathway is aberrantly activated in many types of tumours, most commonly in CRCs (Barker and Clevers, 2000; Polakis, 2000). In addition, up to 38% of metastatic androgen-independent prostate tumours have abnormal accumulation of *β*-catenin (Chesire and Isaacs, 2002; de la Taille *et al*, 2003).

Owing to the nature of HOX proteins manifesting temporal and spatial specification during the developmental stage, some HOX proteins are destined to control the proper development of the posterior part of the body axis. In colorectal tissues, several studies have reported that some HOX proteins, including HOXA5, HOXB6, HOXB8, HOXC8, and HOXC9, are expressed in normal tissues and their deregulation is involved in colorectal tumorigenesis (De Vita *et al*, 1993; Vider *et al*, 2000; Wang *et al*, 2001). Hoxb13 has been shown to be limitedly expressed in the caudal extent of the spinal cord, tail bud and urogenital sinus (Zeltser *et al*, 1996; Sreenath *et al*, 1999). Expression of Hoxb13 in adult mouse is confined to the epithelial cells of the prostate and distal colon and rectum (Zeltser *et al*, 1996; Sreenath *et al*, 1999). Mice homozygous for Hoxb13 loss-of-function mutations showed inhibition of proliferation and activation of programmed cell death in the secondary neural tube and caudal somites (Economides *et al*, 2003). In addition, low HOXB13 expression is known to involve in the skin regeneration and wound healing (Stelnicki *et al*, 1998; Komuves *et al*, 2003; Mack *et al*, 2003). Accordingly, HOXB13 expression was either downregulated or mislocalised in several skin tumours, including melanoma, basal cell carcinoma, and squamous cell carcinoma (Komuves *et al*, 2003; Hoek *et al*, 2004). On the other hand, Ma *et al* (2004) have recently reported that HOXB13 is overexpressed in tamoxifen-resistant, estrogen receptor-positive breast cancers (Ma *et al*, 2004). Ectopic expression of HOXB13 also enhances migration and invasion in breast cancer cells. In this study, we demonstrated that expression of HOXB13 is frequently diminished in colorectal tumours to confer a positive growth signal, which may be accomplished thorough the regulation of *β*-catenin/TCF4 signals.
